# Electrocardiographic Risk Markers of Cardiac Death: Gender Differences in the General Population

**DOI:** 10.3389/fphys.2020.578059

**Published:** 2021-02-05

**Authors:** Mira Anette E. Haukilahti, Tuomas V. Kenttä, Jani T. Tikkanen, Olli Anttonen, Aapo L. Aro, Tuomas Kerola, Antti Eranti, Arttu Holkeri, Harri Rissanen, Markku Heliövaara, Paul Knekt, M. Juhani Junttila, Heikki V. Huikuri

**Affiliations:** ^1^Research Unit of Internal Medicine, Medical Research Center Oulu, University of Oulu and University Hospital of Oulu, Oulu, Finland; ^2^Department of Internal Medicine, Päijät-Häme Central Hospital, Lahti, Finland; ^3^Division of Cardiology, Heart and Lung Center, University of Helsinki and Helsinki University Hospital, Helsinki, Finland; ^4^Heart Center, Central Hospital of North Karelia, Joensuu, Finland; ^5^Department of Public Health Solutions, Finnish National Institute for Health and Welfare (THL), Helsinki, Finland

**Keywords:** gender differences, ECG, sudden cardiac death, cardiac death, left ventricular hypertrophy, prolonged QRS, T wave inversion

## Abstract

**Background:**

Cardiac death is one of the leading causes of death and sudden cardiac death (SCD) is estimated to cause approximately 50% of cardiac deaths. Men have a higher cardiac mortality than women. Consequently, the mechanisms and risk markers of cardiac mortality are not as well defined in women as they are in men.

**Aim:**

The aim of the study was to assess the prognostic value and possible gender differences of SCD risk markers of standard 12-lead electrocardiogram in three large general population samples.

**Methods:**

The standard 12-lead electrocardiographic (ECG) markers were analyzed from three different Finnish general population samples including total of 20,310 subjects (49.9% women, mean age 44.8 ± 8.7 years). The primary endpoint was cardiac death, and SCD and all-cause mortality were secondary endpoints. The interaction effect between women and men was assessed for each ECG variable.

**Results:**

During the follow-up (7.7 ± 1.2 years), a total of 883 deaths occurred (24.5% women, *p* < 0.001). There were 296 cardiac deaths (13.9% women, *p* < 0.001) and 149 SCDs (14.8% women, *p* < 0.001). Among those who had died due to cardiac cause, women had more often a normal electrocardiogram compared to men (39.0 vs. 27.5%, *p* = 0.132). After adjustments with common cardiovascular risk factors and the population sample, the following ECG variables predicted the primary endpoint in men: left ventricular hypertrophy (LVH) with strain pattern (*p* < 0.001), QRS duration > 110 ms (*p* < 0.001), inferior or lateral T-wave inversion (*p* < 0.001) and inferolateral early repolarization (*p* = 0.033). In women none of the variables remained significant predictors of cardiac death in multivariable analysis, but LVH, QTc ≥ 490 ms and T-wave inversions predicted SCD (*p* < 0.047 and 0.033, respectively). In the interaction analysis, LVH (HR: 2.4; 95% CI: 1.2–4.9; *p* = 0.014) was stronger predictor of primary endpoint in women than in men.

**Conclusion:**

Several standard ECG variables provide independent information on the risk of cardiac mortality in men but not in women. LVH and T-wave inversions predict SCD also in women.

## Introduction

Cardiovascular disease (CVD) remains the leading cause of death worldwide both in women and men, despite of the overall reduction in CVD mortality (Naghavi et al., 2015; Maffei et al., 2019). The death rates for CVD have declined 22% from 2005 to 2015 (Benjamin et al., 2018), but they seem now to be again increasing among US women, probably due to obesity epidemic (Mosca et al., 2011). Even though the first female-specific recommendation for prevention of CVD was published by American Heart Association in 1995 (Mosca et al., 1999), the erroneous perception that women are protected against CVD is still strong (Sciomer et al., 2018). Traditional Framingham risk factors for CVD have shown to increase the risk of cardiac mortality in both genders (Greenland et al., 2003) even though the impact of classical risk factors for CVD are likely to also differ among men and women (Maffei et al., 2019). In addition, there are many risk factors unique for women associating to reproductive capability and pregnancy, and the development of CVD in women may correlate with some specific events occurring in their reproductive history (Maffei et al., 2019).

Supplementing the traditional risk assessment with electrocardiographic (ECG) risk markers drawn from standard 12-lead electrocardiogram might assist in detecting subclinical changes in cardiac structure and function in previously asymptomatic subjects. These subjects with elevated risk for CVD could then be referred to risk reduction therapies (O’Malley and Redberg, 2012). In previous literature, ST-segment depression, T-wave inversion, ECG signs of left ventricular hypertrophy (LVH) including strain and premature ventricular contractions have been associated with 2–10-fold risk for coronary artery disease (CAD) events (Chou et al., 2011) and combined cardiovascular events (Jørgensen et al., 2014) among asymptomatic subjects. In addition, recent studies have shown that prolonged QRS duration (Aro et al., 2011), certain patterns of early repolarization (Tikkanen et al., 2009; Junttila et al., 2014) and fragmented QRS complex predict cardiac mortality (Terho et al., 2014). Studies evaluating screening for the risk of cardiac mortality using resting electrocardiogram, focusing especially on gender differences in general populations, are sparse (Holkeri et al., 2020). In the future the lack of awareness of the high CVD risk among certain subgroups of women must be overcome, and better sex-specific risk assessment tools for cardiovascular mortality needs to be developed. The aim of the current study was to assess the prognostic value of ECG risk markers for cardiac mortality but also for SCD and all-cause mortality in three large general population samples and conceive possible gender differences in the prognostic value of these markers.

## Materials and Methods

### Patient Populations

**FIGURE 1:**
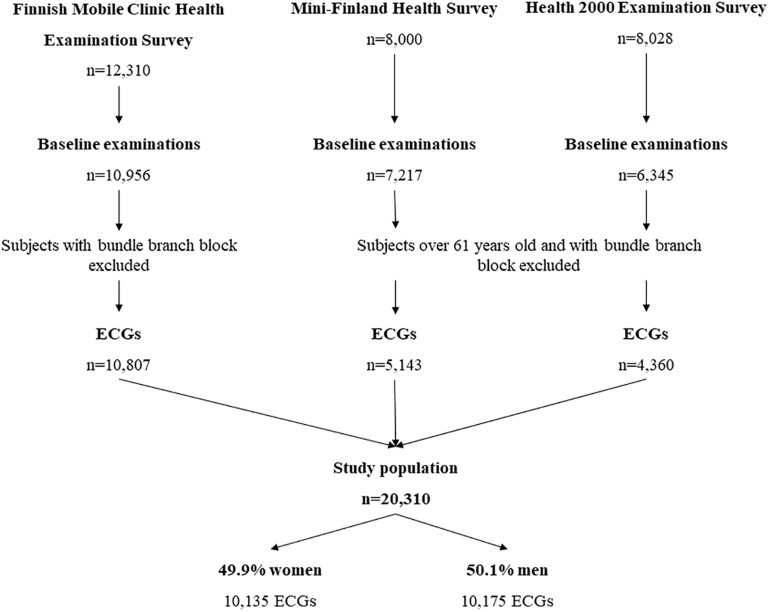
A flowchart of the patient populations.

Standard 12-lead ECGs were analyzed from 20,310 participants (49.9% women, mean age 44.8 ± 8.7 years) of the Coronary Heart Disease Study of the Finnish Mobile Clinic Health Examination Survey (*N* = 10,807), the Mini-Finland Health Survey (*N* = 5,143) and the Health 2000 Health Examination Survey (*N* = 4,360). All three studies are general population-based surveys of middle-aged subjects including the approximately similar number of women and men from different geographical areas in Finland. The studies were conducted in different eras by Social Insurance Institution and National Institution for Health and Welfare. Flowchart in Figure 1 represents the final study population.

#### The Finnish Mobile Clinic Health Examination Survey (1966–1972)

The Coronary Heart Disease Study of the Finnish Mobile Clinic Health Examination Survey consists total of 10,957 subjects aged 30–61 years. The study was carried out between 1966 and 1972 in 4 different geographical areas in Finland. The details of the study population have been described previously (Reunanen et al., 1983). The study subjects underwent recording of standard 12-lead resting electrocardiogram, measurement of blood pressure and body mass index (BMI), and completed a questionnaire regarding their health behavior, known diseases and medications. After excluding 53 subjects with unreadable electrocardiogram and subjects with bundle branch blocks, 10,807 subjects were included in the present study (47.8% female, mean age 44.0 ± 8.5 years).

#### The Mini-Finland Health Survey (1978–1980)

The Mini-Finland Health Survey was conducted between 1978 and 1980, and the primary study population of 8,000 subjects aged ≥ 30 years and was representative of the Finnish population at the time. Out of these subjects, 7,217 participated in similar health examinations and measurements as in Mobile Clinic Study above. The detailed descriptions of the study protocol have been published elsewhere (Knekt et al., 2017). In the current study, subjects older than 61 years were excluded to standardize the study samples. After exclusion, a total of 5,143 subjects (52.2% female, mean age 44.6 ± 9.2 years) remained in the final study population sample.

#### The Health 2000 Survey (2000–2001)

The Health 2000 Health Examination Survey was carried out between 2000 and 2001. The primary study population contained a representative sample of 8,028 adults between ages 30 and 80. The baseline examinations were conducted on 6,345 subjects and after excluding unreadable electrocardiograms 6,305 subjects remained (54.6% female, mean age 51.5 ± 12.8 years). The details of study population have been described previously (Heistaro, 2020). In the current study, subjects over 61 years old were excluded to standardize the study samples. After exclusions, a total of 4,360 subjects (52.3% female, mean age 45.5 ± 8.4 years) remained in the final study population sample.

### Electrocardiography

The standard resting 12-lead electrocardiograms were recorded at paper speed of 50 mm/s and calibration of 10 mm/mV at study baseline. QRS duration and QT interval were measured from leads II or V5. QRS duration was considered prolonged if it was ≥ 110 ms. Heart rate correction for the QT interval was performed with Bazett’s formula. The presence of early repolarization ≥ 0.1 mV with horizontal or descending ST segment and fragmented QRS were assessed as described previously (Tikkanen et al., 2009; Terho et al., 2014). Early repolarization was classified inferolateral if there were ≥ 2 slurred or notched J-point elevations ≥ 0.1 mV in inferior (II, III, aVF) or lateral (I, aVL, V4–V6) leads. ST segment was classified as horizontal or descending if it was under 0.1 mV 100 ms after the end of the QRS complex. Fragmented QRS complexes were classified by coronary artery regions as anterior (V1–V3), inferior or lateral if there were ≥ 2 fragmented QRS complexes within the region. Similarly, inferolateral T-wave inversions were classified by coronary artery regions as inferior or lateral if T-wave amplitude was < −0.1 mV in ≥ 2 contiguous leads in the same region. The Sokolow-Lyon criteria was used for grading electrocardiographic LVH, i.e., if the sum of S wave amplitude in V1 and R wave amplitude in V5/V6 (whichever larger) was ≥35 mm, electrocardiogram was graded as positive for LVH. By LVH with repolarization abnormalities we refer to ECG sign of LVH observed with inferior or lateral T-wave inversions. Any ECG abnormality variable indicated the presence of at least one of the following ECG risk markers: QRS duration ≥ 110 ms, QTc ≥ 450 ms in men, or ≥470 ms in women, presence of ECG signs of LVH, T-wave inversion, inferolateral ER or fQRS. Prevalence and risks for endpoints of any other ECG abnormality was not assessed in this study. Subjects with a bundle branch block were excluded from the final study population.

### Follow-Up and Endpoints

In the Coronary Heart Disease Study of the Finnish Mobile Clinic Health Examination Survey, baseline measurements were performed 1966-1972. The participants were followed for 30 ± 11 years until the end of year 2007. In the Mini-Finland Study the baseline measurements were performed 1978–1980 and participants were followed for 24.0 ± 10.6 years from the baseline examinations until the end of 2011. The baseline measurements of the Health 2000 Study were carried out in 2000 and 2001. The mean follow-up time was 8.8 ± 1.1 years until the end of January 2009. In the present analyses the follow-up time was limited to 8 years in all analyses to clarify the role of electrocardiography in the risk assessment since the cardiovascular risk profile could ultimately change during a longer follow-up period.

The primary endpoint of the study was cardiac death and the secondary endpoints were sudden cardiac death and death from any cause. The causes of death were determined using nationwide health registers; Causes of Death Register maintained by Statistics Finland. The quality and validity of these registers has been well established (Rapola et al., 1997). Death of cardiac cause was defined using International Classification of Disease code of cause of death representing the codes I20–I25 in the International Classification of Diseases 10. A committee of qualified and experienced cardiologists unaware of the ECG analysis reviewed all the cardiac deaths. They evaluated each case by using the death certificates and hospital records. Definition used for SCD is based on definitions presented in Cardiac Arrhythmia Pilot Study and has been described earlier in detail (Greene et al., 1989; Tikkanen et al., 2009). In addition, death was classified as SCD if the event was unwitnessed yet lacking the evidence of other cause of death. All three studies were approved by the local ethics committees and followed the guidelines of The Declaration of Helsinki.

### Statistical Analyses

All continuous variables are presented as mean ± standard deviation (SD) and categorical variables as number of cases with prevalence among the study population in brackets. When comparing the categorial variables between groups of interest the Pearson Chi-Square test was used. Similarly, Student’s *t*-test was used when comparing continuous variables. Cox proportional hazards model was used to calculate hazard ratios (HR) and their 95% confidence intervals (CI) in the pooled data. In multivariable analysis, variables were adjusted by age, smoking, diabetes, CAD, BMI, cholesterol, systolic blood pressure, heart rate, and the population sample. The statistical significance of the interaction effect between gender and prognostic value of each ECG variable was tested using the Cox regression analysis. In addition, Cox regression was applied separately for each cohort and the subsequent results were analyzed via random effects meta-analysis model in order to get the pooled hazard ratios and heterogeneity analyses (Cochrane’s Q). For Cochranes Q, *P* < 0.1 was considered to represent statistically significant heterogeneity between populations. All statistical analyses were performed using Statistical Package for Social Studies version 26.0 (IBM SPSS Statistics) and R version 3.6.3 (R Foundation for Statistical Computing, Vienna, Austria.). We considered *p*-value of < 0.05 as statistically significant.

## Results

The standard 12-lead ECG markers were analyzed from 20,310 subjects (49.9% women, mean age 44.8 ± 8.7 years). Characteristics of the study population are represented in Table 1. On average women were slightly older at the time of baseline measurements in comparison to men, and they also had higher heart rate and longer QTc duration than men. Men had longer QRS complexes than women. There were considerably more smokers among men than in women in each study population combined, and men also had on average higher prevalence of diabetes mellitus. Prevalence for each end point were considerably higher among male subjects in comparison to female subjects. Approximately two fifth of the subjects who experienced cardiac death (40.6%, *N* = 317, *p* < 0.001) or SCD (40.0%, *N* = 120, *p* < 0.001) had CAD. The proportion of CAD was expectedly higher among male victims: 44.7 vs. 33.2% for men and women, (respectively) among subjects with cardiac death (*p* < 0.001), and 43.8 vs. 28.4% among victims of SCD (*p* < 0.001).

**TABLE 1:** The baseline characteristics of each population sample.

	Finnish mobile clinic health examination survey, *N* = 10,807		Mini-finland health survey, *N* = 5,143		Health 2000 examination survey, *N* = 4,360	
			
	Female *N* = 5,167	Male *N* = 5,640	Female *N* = 2,686	Male *N* = 2,457	Female *N* = 2,282	Male *N* = 2,078
Continuous variables	Mean ± SD	Mean ± SD	Mean ± SD	Mean ± SD	Mean ± SD	Mean ± SD
Age (years)	44.3 ± 8.4	43.7 ± 8.5	44.9 ± 9.3	44.4 ± 9.1	45.4 ± 8.5	45.6 ± 8.4
BMI (kg/m^2^)	26.2 ± 4.4	25.6 ± 3.3	25.5 ± 4.5	25.8 ± 3.5	26.1 ± 5.0	27.0 ± 4.2
Systolic blood pressure (mmHg)	138.1 ± 23.5	138.7 ± 19.5	137.9 ± 20.9	139.8 ± 18.2	125.9 ± 18.4	131.6 ± 16.6
Cholesterol (mmol/l)	6.5 ± 1.3	6.5 ± 1.3	3.9 ± 1.1	4.5 ± 1.3	5.7 ± 1.1	6.0 ± 1.1
QRS duration (ms)	84.8 ± 7.0	88.4 ± 7.8	82.6 ± 7.8	89.4 ± 8.8	86.5 ± 8.4	95.5 ± 10.1
QTc (ms)	415.2 ± 26.2	401.7 ± 27.1	419.5 ± 23.7	405.3 ± 23.8	416.9 ± 15.9	410.9 ± 15.6
Heart rate (1/min)	79.6 ± 15.5	72.0 ± 14.2	69.0 ± 12.7	64.7 ± 12.8	63.8 ± 10.3	62.3 ± 11.1

**Categorial variables**	**N (%)**	**N (%)**	**N (%)**	**N (%)**	**N (%)**	**N (%)**

QRS ≥ 110 ms	7 (0.1%)	59 (1.0%)	6 (0.2%)	42 (1.7%)	13 (0.6%)	166 (8.0%)
QTc ≥ 470 ms (W)/ ≥ 450 ms (M)	107 (2.1%)	232 (4.1%)	51 (1.9%)	75 (3.1%)	17 (0.7%)	31 (1.5%)
QTc ≥ 490 ms	16 (0.3%)	14 (0.2%)	14 (0.5%)	5 (0.2%)	2 (0.1%)	1 (0.0%)
LVH	1,058 (20.5%)	2,465 (43.8%)	139 (5.2%)	467 (19.0%)	76 (3.3%)	275 (13.2%)
LVH + T-wave inversion	6 (0.1%)	26 (0.5%)	8 (0.3%)	20 (0.8%)	3 (0.1%)	2 (0.1%)
Inferior or lateral T-wave inversion	29 (0.6%)	45 (0.8%)	31 (1.2%)	36 (1.5%)	6 (0.3%)	7 (0.3%)
Inferolateral ER	224 (4.3%)	406 (7.2%)	361 (13.4%)	472 (19.2%)	35 (1.5%)	130 (6.3%)
Lateral ER	111 (2.2%)	151 (2.8%)	194 (7.2%)	183 (7.4%)	28 (1.2%)	101 (4.9%)
Inferior ER	114 (2.3%)	270 (4.9%)	174 (6.5%)	313 (12.7%)	10 (0.4%)	50 (2.4%)
Inferolateral ER + horizontal/descending ST-segment	175 (3.4%)	225 (4.1%)	238 (8.9%)	295 (12.0%)	22 (1.0%)	56 (2.7%)
Any fQRS	778 (15.2%)	1,196 (21.4%)	465 (17.3%)	418 (17.0%)	1,324 (58.2%)	1,392 (68.6%)
Lateral fQRS	30 (0.7%)	52 (1.2%)	48 (1.8%)	48 (2.0%)	818 (36.0%)	1,050 (51.7%)
Inferior fQRS	650 (13.0%)	1,062 (19.5%)	375 (14.0%)	327 (13.3%)	701 (30.8%)	592 (29.2%)
Anterior fQRS	141 (3.1%)	173 (3.8%)	65 (2.4%)	68 (2.8%)	163 (7.2%)	146 (7.2%)
Smoking	680 (13.2%)	2,990 (53.0%)	431 (16.0%)	964 (39.2%)	502 (22.1%)	653 (31.5%)
Diabetes	82 (1.6%)	111 (2.0%)	58 (2.2%)	90 (3.7%)	54 (2.4%)	103 (5.0%)
CAD	753 (14.6%)	714 (12.7%)	110 (4.1%)	156 (6.3%)	13 (0.6%)	56 (2.7%)
Cardiac death	22 (0.4%)	154 (2.7%)	12 (0.4%)	73 (3.0%)	7 (0.3%)	28 (1.3%)
Sudden cardiac death	12 (0.2%)	73 (1.3%)	6 (0.2%)	26 (1.1%)	4 (0.2%)	28 (1.4%)
All-cause mortality	115 (2.2%)	408 (7.2%)	63 (2.3%)	174 (7.1%)	38 (1.7%)	85 (4.1%)

**For categorical variables values are presented as number of cases within genders and corresponding percentage within brackets. Continuous values are presented as mean with standard deviation (SD) below to it. Underlined values differed statistically significantly (p < 0.05) between genders. BMI, body mass index; CAD, coronary artery disease; SD, standard deviation.**

During the follow-up of 7.7 ± 1.2 years, a total of 296 cardiac deaths occurred of which 13.9% were among female subjects (*N* = 41, *p* < 0.001). The prevalence of different ECG markers is represented in Table 2. At least one ECG abnormality was present in 61.0% of the female victims of cardiac death. Only prevalence of fQRS was higher among women than in men, though the number of cases was so low that no statistically significant differences in the prevalence of ECG abnormalities between genders were detected. LVH with and without repolarization abnormalities, QTc prolongation > 470 ms and inferior or lateral T-wave inversions associated with the relative risk for cardiac death among women in univariate model but did not remain statistically significant after adjustments with age, smoking, diabetes, CAD, BMI, cholesterol, systolic blood pressure, heart rate, and the study sample (Table 2). Only extremely prolonged QTc time associated with higher risk for cardiac death among women than in men, but this ECG marker was found only from one woman. In random effects meta-analysis any of the ECG abnormalities were not statistically significant in women. However, statistically significant interaction effect was observed between gender and prognostic value of LVH (Table 3), and it provided stronger prognostic value for cardiac death in women than in men.

**TABLE 2:** The gender differences in the prevalence and risks for cardiac death of each ECG risk marker with 8 years follow-up time.

	Women, *N* = 41					Men, *N* = 255				
		
	Unadjusted			Adjusted		Unadjusted			Adjusted	
				
	N (%)	HR (95% CI)	*P*-value	HR (95% CI)	*P*-value	N (%)	HR (95% CI)	*P*-value	HR (95% CI)	*P*-value
Any ECG abnormality	25 (61.0%)	**2.2 (1.2**–**4.2)**	**0.012**	1.6 (0.80–3.1)	0.185	182 (72.5%)	**1.7 (1.3**–**2.2)**	**<0.001**	**1.6 (1.2**–**2.1)**	**0.002**
QRS ≥ 110 ms	0 (0.0%)	No events		No events		14 (5.5%)	**2.2 (1.3**–**3.8)**	**0.004**	**3.1 (1.8**–**5.3)**	**<0.001**
QTc ≥ 470 ms (W)/ ≥ 450 ms (M)	3 (7.3%)	**4.6 (1.4**–**14.9)**	**0.011**	3.2 (0.95–10.9)	0.061	23 (9.2%)	**3.1 (2.0**–**4.7)**	**<0.001**	1.3 (0.80–2.0)	0.306
QTc ≥ 490 ms	**1 (2.4%)**	**8.0 (1.1–58.1)**	**0.040**	**9.4 (1.2–71.7)**	**0.031**	1 (0.4%)	2.5 (0.35–17.9)	0.357	1.4 (0.19–10.2)	0.737
LVH	13 (31.7%)	**3.3 (1.7**–**6.3)**	**<0.001**	1.8 (0.79–3.9)	0.167	97 (38.2%)	**1.4 (1.1**–**1.7)**	**<0.019**	1.2 (0.90–1.6)	0.209
LVH + T-wave inversion	2 (4.9%)	**38.5 (9.3**–**159.4)**	**<0.001**	3.2 (0.40–25.3)	0.275	13 (5.1%)	**17.0 (9.7**–**29.8)**	**<0.001**	**3.4 (1.9**–**6.2)**	**<0.001**
Inferior or lateral T-wave inversion	3 (7.3%)	**12.9 (4.0**–**41.7)**	**<0.001**	2.0 (0.47–8.8)	0.341	26 (10.2%)	**17.8 (11.8**–**26.7)**	**<0.001**	**4.3 (2.8**–**6.7)**	**<0.001**
Inferolateral ER	2 (4.9%)	0.79 (0.19–3.3)	0.748	0.64 (0.15–2.7)	0.542	31 (12.2%)	1.3 (0.87–1.8)	0.218	1.1 (0.78–1.7)	0.438
Lateral ER	1 (2.4%)	0.74 (0.10–5.4)	0.766	0.54 (0.07–4.1)	0.553	11 (4.4%)	1.0 (0.55–1.8)	0.978	1.0 (0.56–1.9)	0.904
Inferior ER	1 (2.5%)	0.84 (0.12–6.1)	0.859	0.77 (0.10–5.8)	0.799	22 (8.8%)	1.5 (0.94–2.3)	0.091	1.3 (0.80–2.0)	0.326
Inferolateral ER + horizontal/descending ST-segment	2 (4.9%)	1.2 (0.28–4.8)	0.847	1.6 (0.38–6.9)	0.511	19 (7.6%)	1.4 (0.86–2.2)	0.192	**1.7 (1.0**–**2.7)**	**0.033**
Any fQRS	14 (34.1%)	1.5 (0.80–2.9)	0.197	1.6 (0.78–3.3)	0.197	77 (31.0%)	1.1 (0.81–1.4)	0.685	1.3 (0.97–1.7)	0.079
Lateral fQRS	6 (16.2%)	1.8 (0.77–4.4)	0.171	2.4 (0.64–9.3)	0.188	25 (11.5%)	0.88 (0.56–1.3)	0.537	1.7 (0.99–3.0)	0.054
Inferior fQRS	8 (20.0%)	1.2 (0.55–2.6)	0.641	1.1 (0.49–2.6)	0.777	50 (20.8%)	1.1 (0.77–1.4)	0.725	1.2 (0.86–1.62)	0.300
Anterior fQRS	3 (8.3%)	2.2 (0.69–7.3)	0.181	2.3 (0.69–7.7)	0.175	13 (6.0%)	1.4 (0.82–2.5)	0.195	1.3 (0.70–2.2)	0.464

**CI, confidence interval; ER, early repolarization; fQRS, fragmented QRS; HR, hazard ratio; LVH, left ventricular hypertrophy. Results are adjusted by age, smoking, diabetes, CAD, BMI, cholesterol, systolic blood pressure, heart rate, and the study sample. Statistically significant values p < 0.05 are written in bold.**

**TABLE 3:** The interaction effect between sex and the prognostic value of each ECG variable.

	Women vs. Men								

	Cardiac death			SCD			All-cause mortality		
			
	HR	95% CI	*P*-value	HR	95% CI	*P*-value	HR	95% CI	*P*-value
Any ECG abnormality	1.3	0.67–2.6	0.419	1.4	0.51–3.7	0.532	**1.7**	**1.2**–**2.3**	**0.001**
QRS ≥ 110 ms	No events in women			No events in women			1.2	0.16–8.8	0.868
QTc ≥ 470 ms (W)/ ≥ 450 ms (M)	1.5	0.43–5.2	0.530	2.0	0.42–9.8	0.384	0.91	0.44–1.9	0.914
QTc ≥ 490 ms	0.32	0.02–5.2	0.420	0.33	0.02–5.4	0.437	1.9	0.39–9.7	0.420
Inferior or lateral T-wave inversion	0.72	0.21–2.5	0.600	0.92	0.19–4.4	0.913	0.81	0.40–1.7	0.561
LVH	**2.4**	**1.2**–**4.9**	**0.014**	2.5	0.99–6.4	0.053	**1.7**	**1.2**–**2.4**	**0.003**
LVH + T-wave inversion	2.2	0.48–10.1	0.311	1.6	0.20–13.8	0.649	2.3	0.95–5.4	0.064
Inferolateral ER	0.63	0.14–2.7	0.531	0.85	0.10–6.9	0.877	1.6	0.98–2.6	0.059
Lateral ER	0.73	0.09–5.8	0.768		No events		**3.1**	**1.6**–**5.7**	**<0.001**
Inferior ER	1.1	0.13–8.9	0.938	0.93	0.09–9.2	0.949	0.73	0.31–1.7	0.482
Inferolateral ER + horizontal/descending ST-segment	0.84	0.19–3.8	0.821	1.3	0.15–11.2	0.824	1.2	0.65–2.2	0.548
Any fQRS	1.4	0.72–2.9	0.301	1.0	0.40–2.8	0.923	**1.6**	**1.1**–**2.2**	**0.009**
Lateral fQRS	2.1	0.79–5.5	0.133	0.71	0.97–2.6	0.064	**1.8**	**1.1**–**3.0**	**0.014**
Inferior fQRS	1.1	0.49–2.6	0.764	1.1	0.33–3.4	0.919	0.82	no events	0.986
Anterior fQRS	1.5	0.42–5.7	0.514	0.97	0.11–8.6	0.980	**1.8**	0.35–1.9	0.647

*Interactions between gender and ECG risk markers were assessed using Cox regression analysis.*

*CI, confidence interval; ER, early repolarization; fQRS, fragmented QRS; HR, hazard ratio; LVH, left ventricular hypertrophy; SCD, sudden cardiac death. HR, hazard ratio. Statistically significant values (p < 0.05) are written in bold.*

Among male subjects who had died for cardiac causes at least one ECG abnormality was present in 72.5%. The prevalence of each ECG variable, excluding fQRS, was slightly higher among male cardiac death victims in comparison to female cardiac death victims. Among men prolonged QRS duration, LVH with strain changes, inferior or lateral T-wave inversions and inferolateral ER predicted the occurrence of cardiac death in multivariate model (Table 2). In random effects meta-analysis prolonged QRS [HR: 3.0 (95% CI: 1.7–5.2, *P* < 0.001), Cochrane *Q*-value: 1.1 (*P* = 0.582)] and inferior or lateral T-wave inversion [HR: 3.8 (95% CI: 2.0–7.0, *P* < 0.001), Cochrane *Q*-value: 2.5 (*P* = 0.293)] remained statistically significant in competing risks regression multivariable model and associated with cardiac death, as well as any ECG abnormality [HR: 1.6 (95% CI: 1.0–2.4, *P* = 0.035), Cochrane *Q*-value: 3.0 (*P* = 0.229)].

The prognostic value of ECG variables for SCD and all-cause mortality are shown in Tables 4, 5. T-wave inversions, extremely prolonged QTc and LVH remained statistically significant predictors for SCD in multivariable analysis both in women and in men. In men, T-wave inversion [HR: 3.2 (95% CI: 1.7–6.1, *P* = 0.001), Cochrane *Q*-value: 1.9 (*P* = 0.386)] and LVH [HR: 1.6 (95% CI: 1.1–2.4, *P* = 0.025), Cochrane *Q*-value: 0.158 (*P* = 0.924)] remained statistically significant risk factors for SCD also in random effect meta-analysis as did any ECG abnormality [HR: 1.7 (95% CI: 1.1–2.7, *P* = 0.011), Cochrane *Q*-value: 0.630 (*P* = 0.730)] and prolonged QRS duration [HR: 3.4 (95% CI: 1.8–6.4, *P* < 0.001), Cochrane *Q*-value: 1.1 (*P* = 0.573)]. Any of the ECG variables were not statistically significant among women in random effect meta-analysis, and no statistically significant interaction between gender and the ECG variables were seen for SCD. LVH, prolonged QRS duration and any ECG abnormality associated with moderately higher risk of SCD among subjects with prior CAD. LVH with repolarization abnormalities and T wave inversions associated with considerably higher risk among SCD victims without prior CAD but number of cases was substantially low. Differences among SCD victims with and without prior CAD are represented in Table 6. For all-cause mortality statistically significant interactions between gender and LVH, lateral ER, total and lateral fQRS as well as any ECG abnormality were seen, the abnormalities having greater prognostic value in women than in men.

**TABLE 4:** The gender differences in the prevalence and risks for sudden cardiac death of each ECG risk marker with 8 years follow-up time.

	Women, *N* = 22					Men, *N* = 127				
		
	Unadjusted			Adjusted		Unadjusted			Adjusted	
				
	N (%)	HR (95% CI)	*P*-value	HR (95%CI)	*P*-value	N (%)	HR (95% CI)	*P*-value	HR (95% CI)	*P*-value
Any ECG abnormality	15 (68.2%)	**3.1 (1.3**–**7.5)**	**0.014**	2.3 (0.90–5.8)	0.084	98 (77.8%)	**2.2 (1.5**–**3.4)**	**<0.001**	**1.9 (1.3**–**2.9)**	**0.003**
QRS ≥ 110 ms	0 (0.0%)	No events		No events		11 (8.7%)	**3.6 (1.9**–**6.7)**	**<0.001**	**3.7 (1.9**–**7.0)**	**<0.001**
QTc ≥ 470 ms (W)/ ≥ 450 ms (M)	2 (9.1%)	**5.8 (1.4**–**24.9)**	**0.018**	4.2 (0.93–19.0)	0.063	11 (8.7%)	**2.9 (1.5**–**5.3)**	**0.001**	1.3 (0.67–2.5)	0.437
QTc ≥ 490 ms	**1 (4.5%)**	**15.2 (2.0–113.2)**	**0.008**	**14.8 (1.8–119.1)**	**0.011**	1 (0.8%)	4.9 (0.69–35.3)	0.112	**2.4 (1.0–5.4)**	**0.039**
Inferior or lateral T-wave inversion	2 (9.1%)	**16.3 (3.8**–**69.8)**	**<0.001**	**5.4 (1.1**–**25.6)**	**0.033**	13 (10.2%)	**17.5 (9.9**–**31.1)**	**<0.001**	**4.6 (2.5**–**8.5)**	**<0.001**
LVH	8 (36.4%)	**4.0 (1.7**–**9.6)**	**0.002**	**2.8 (1.0**–**7.8)**	**0.047**	53 (42.1%)	**1.6 (1.1**–**2.3)**	**0.010**	**1.7 (1.2**–**2.6)**	**0.008**
LVH + T-wave inversion	1 (4.5%)	**36.1 (4.8**–**268.3)**	**<0.001**	8.0 (0.88–71.9)	0.064	8 (6.3%)	**21.0 (10.3**–**43.1)**	**<0.001**	**5.3 (2.4**–**11.4)**	**<0.001**
Inferolateral ER	1 (4.5%)	0.74 (0.10–5.5)	0.765	0.61 (0.08–4.8)	0.639	11 (8.7%)	0.87 (0.47–1.6)	0.651	0.90 (0.48–1.7)	0.732
Lateral ER	0 (0.0%)	No events		No events		1 (0.8%)	0.18 (0.03–1.3)	0.084	0.20 (0.03–1.4)	0.104
Inferior ER	1 (4.5%)	1.6 (0.21–11.5)	0.668	1.6 (0.20–12.6)	0.658	11 (8.7%)	1.4 (0.77–2.6)	0.261	1.5 (0.78–2.8)	0.239
Inferolateral ER + horizontal/descending ST-segment	1 (4.5%)	1.1 (0.14–7.9)	0.949	1.5 (0.20–11.4)	0.699	6 (4.8%)	0.83 (0.37–1.9)	0.663	1.1 (0.48–2.5)	0.819
Any fQRS	7 (31.8%)	1.4 (0.56–3.4)	0.485	1.4 (0.52–3.7)	0.504	43 (35.8%)	1.3 (0.90–1.9)	0.154	1.3 (0.84–1.9)	0.270
Lateral fQRS	2 (10.5%)	1.1 (0.26–4.8)	0.880	0.98 (0.16–6.1)	0.986	20 (19.0%)	1.6 (0.97–2.6)	0.064	1.7 (0.86–3.3)	0.127
Inferior fQRS	4 (18.2%)	1.1 (0.36–3.2)	0.904	1.1 (0.36–3.2)	0.906	23 (20.0%)	1.0 (0.64–1.6)	0.984	1.0 (0.66–1.6)	0.866
Anterior fQRS	1 (5.3%)	1.4 (0.18–10.3)	0.760	1.3 (0.16–9.7)	0.828	6 (5.9%)	1.4 (0.62–3.2)	0.417	1.1 (0.48–2.5)	0.815

**CI, confidence interval; ER, early repolarization; fQRS, fragmented QRS; HR, hazard ratio; LVH, left ventricular hypertrophy. Results are adjusted by age, smoking, diabetes, CAD, BMI, cholesterol, systolic blood pressure, heart rate, and the study sample. Statistically significant values (p < 0.05) are written in bold.**

**TABLE 5:** The gender differences in the prevalence and risks for all-cause mortality of each ECG risk marker with 8 years follow-up time.

	Women, *N* = 216					Men, *N* = 667				
		
	Unadjusted			Adjusted		Unadjusted			Adjusted	
				
	N (%)	HR (95% CI)	*P*-value	HR (95% CI)	*P*-value	N (%)	HR (95% CI)	*P*-value	HR (95% CI)	*P*-value
Any ECG abnormality	131 (60.9%)	**2.2 (1.7**–**2.9)**	**<0.001**	**1.8 (1.4**–**2.4)**	**<0.001**	445 (67.5%)	**1.3 (1.1**–**1.6)**	**0.001**	**1.2 (1.0**–**1.5)**	**0.012**
QRS ≥ 110 ms	**1 (0.5%)**	1.8 (0.26–13.0)	0.548	1.7 (0.24–12.1)	0.601	**26 (3.9%)**	**1.5 (1.0**–**2.3)**	**0.032**	**1.9 (1.3**–**2.8)**	**0.002**
QTc ≥ 470 ms (W)/ ≥ 450 ms (M)	**9 (4.2%)**	**2.5 (1.3**–**4.9)**	**0.006**	1.6 (0.81–3.1)	0.182	**55 (8.4%)**	**2.8 (2.1**–**3.6)**	**<0.001**	1.3 (0.96–1.7)	0.090
QTc ≥ 490 ms	2 (0.9%)	3.0 (0.74–12.0)	0.123	1.7 (0.42–7.1)	0.444	**6 (0.9%)**	**5.8 (2.6–12.9)**	**<0.001**	**2.4 (1.0–5.4)**	**0.039**
Inferior or lateral T-wave inversion	10 (4.6%)	**7.9 (4.2**–**14.9)**	**<0.001**	**2.7 (1.4**–**5.5)**	**0.005**	39 (5.8%)	**9.7 (7.0**–**13.4)**	**<0.001**	**3.1 (2.2**–**4.4)**	**<0.001**
LVH	**50 (23.1%)**	**4.0 (1.7**–**9.6)**	**0.002**	**1.5 (1.0**–**2.1)**	**0.032**	**239 (36.0%)**	**1.6 (1.1**–**2.3)**	**0.010**	1.1 (0.91–1.3)	0.369
LVH + T-wave inversion	7 (3.2%)	**24.9 (11.7**–**52.8)**	**<0.001**	**7.4 (3.1**–**17.3)**	**<0.001**	22 (3.3%)	**10.8 (7.1**–**16.5)**	**<0.001**	**3.0 (1.9**–**4.8)**	**<0.001**
Inferolateral ER	23 (10.6%)	**1.8 (1.2**–**2.8)**	**0.006**	1.5 (0.98–2.4)	0.062	74 (11.1%)	1.1 (0.90–1.5)	0.283	1.1 (0.84–1.4)	0.543
Lateral ER	**18 (8.3%)**	**2.7 (1.7**–**4.4)**	**<0.001**	**2.1 (1.3**–**3.5)**	**0.003**	**25 (3.9%)**	0.88 (0.59–1.3)	0.521	0.92 (0.61–1.4)	0.673
Inferior ER	**6 (2.9%)**	0.96 (0.43–2.2)	0.917	0.84 (0.37–1.9)	0.674	**52 (8.0%)**	1.3 (0.98–1.7)	0.067	1.2 (1.90–1.6)	0.227
Inferolateral ER + horizontal/descending ST-segment	14 (6.5%)	1.6 (0.90–2.7)	0.111	**1.8 (1.0**–**3.1)**	**0.039**	47 (7.2%)	1.3 (0.96–1.7)	0.098	**1.5 (1.1**–**2.1)**	**0.007**
Any fQRS	71 (27.5%)	**1.5 (1.1**–**1.9)**	**0.010**	**1.5 (1.1**–**2.1)**	**0.007**	187 (28.5%)	0.94 (0.79–1.1)	0.448	1.1 (0.91–1.3)	0.374
Lateral fQRS	27 (14.2%)	**1.6 (1.0**–**2.4)**	**0.029**	**2.0 (1.1**–**3.4)**	**0.017**	66 (11.3%)	0.86 (0.67–1.1)	0.256	**1.5 (1.1**–**2.1)**	**0.025**
Inferior fQRS	50 (23.5%)	**1.5 (1.1**–**2.0)**	**0.016**	**1.5 (1.1**–**2.1)**	**0.018**	127 (19.7%)	0.99 (0.81–1.2)	0.897	1.1 (0.87–1.3)	0.582
Anterior fQRS	7 (3.7%)	0.94 (0.44–2.0)	0.866	0.94 (0.44–2.0)	0.881	28 (4.8%)	1.1 (0.78–1.7)	0.494	1.1 (0.72–1.6)	0.763

**CI, confidence interval; ER, early repolarization; fQRS, fragmented QRS; HR, hazard ratio; LVH, left ventricular hypertrophy. Statistically significant values (p < 0.05) are written in bold. Results are adjusted by age, smoking, diabetes, CAD, BMI, cholesterol, systolic blood pressure, heart rate, and the study sample.**

**TABLE 6:** Differences in the prevalence and risks for sudden cardiac death among subjects with and without previous coronary artery disease.

	SCD victims without prior CAD, *N* = 93					SCD victims with prior CAD, *N* = 56				
		
	Unadjusted			Adjusted		Unadjusted			Adjusted	
				
	N (%)	HR (95% CI)	*P*-value	HR (95%CI)	*P*-value	N (%)	HR (95% CI)	*P*-value	HR (95% CI)	*P*-value
Any ECG abnormality	66 (71.7%)	**2.5 (1.6–3.9)**	**<0.001**	**1.9 (1.2–3.0)**	**0.009**	47 (83.9%)	**4.0 (2.0–8.2)**	**<0.001**	**3.6 (1.7–7.4)**	**0.001**
QRS ≥ 110 ms	**6 (6.5%)**	**5.0 (2.2–11.3)**	**<0.001**	**3.7 (1.6–8.7)**	**0.002**	**5 (8.9%)**	**5.5 (2.2–13.9)**	**<0.001**	**7.5 (2.8–19.7)**	**<0.001**
QTc ≥ 470 ms (W)/ ≥ 450 ms (M)	6 (6.5%)	**3.1 (1.4–7.2)**	**0.007**	1.7 (0.71–4.0)	0.233	7 (12.5%)	**2.4 (1.1–5.3)**	**0.031**	2.0 (0.88–4.7)	0.098
QTc ≥ 490 ms	1 (1.1%)	5.0 (0.69–35.7)	0.111	3.0 (0.40–21.9)	0.286	1 (1.8%)	4.0 (0.56–29.1)	0.168	4.0 (0.51–32.1)	0.188
Inferior or lateral T-wave inversion	7 (7.5%)	**27.5 (12.7–59.4)**	**<0.001**	**15.8 (6.8–36.7**)	**<0.001**	8 (14.3%)	**3.7 (1.7–7.8)**	**0.001**	**2.8 (1.3–6.1)**	**0.009**
LVH	32 (34.4%)	**2.0 (1.3–3.1)**	**0.002**	**1.9 (1.2–3.1)**	**0.009**	29 (52.7%)	**2.3 (1.4–3.9)**	**0.002**	**2.7 (1.5–4.8)**	**0.001**
LVH + T-wave inversion	**2 (2.2%)**	**25.0 (6.1–101.4)**	**<0.001**	**9.3 (2.1–41.1)**	**0.003**	**7 (12.7%)**	**8.0 (3.6–17.7)**	**<0.001**	**6.1 (2.5–14.6)**	**<0.001**
Inferolateral ER	6 (6.5%)	0.81 (0.35–1.8)	0.615	0.82 (0.35–1.9)	0.644	6 (10.7%)	1.2 (0.53–2.9)	0.623	1.2 (0.49–2.7)	0.748
Lateral ER	1 (1.1%)	0.28 (0.04–2.0)	0.201	0.29 (0.04–2.1)	0.224	0 (0.0%)	No events		No events	
Inferior ER	6 (6.5%)	1.5 (0.64–3.3)	0.367	1.5 (0.65–3.5)	0.333	6 (10.7%)	2.1 (0.88–4.8)	0.096	1.8 (0.75–4.3)	0.188
Inferolateral ER + horizontal/descending ST-segment	4 (4.3%)	0.88 (0.32–2.4)	0.793	1.3 (0.47–3.5)	0.629	3 (5.4%)	1.0 (0.33–3.3)	0.940	1.1 (0.34–3.5)	0.897
Any fQRS	33 (36.7%)	1.5 (0.97–2.3)	0.072	1.2 (0.74–2.0)	0.437	17 (32.7%)	1.8 (0.99–3.2)	0.053	1.5 (0.82–2.7)	0.195
Lateral fQRS	17 (20.2%)	**1.9 (1.1–3.3)**	**0.017**	1.5 (0.71–3.0)	0.307	5 (12.5%)	**2.9 (1.1–7.5)**	**0.025**	2.7 (0.96–7.8)	0.059
Inferior fQRS	15 (16.9%)	0.88 (0.50–1.5)	0.645	0.83 (0.48–1.5)	0.523	12 (25.0%)	1.7 (0.90–3.3)	0.101	1.6 (0.81–3.0)	0.190
Anterior fQRS	5 (6.0%)	1.5 (0.62–3.8)	0.349	1.3 (0.53–3.3)	0.541	2 (5.3%)	1.1 (0.26–4.4)	0.930	0.77 (0.18–3.2)	0.716

**CAD, coronary artery disease; CI, confidence interval; ER, early repolarization; fQRS, fragmented QRS; HR, hazard ratio; LVH, left ventricular hypertrophy. Results are adjusted by age, smoking, diabetes, CAD, BMI, cholesterol, systolic blood pressure, heart rate, medication for hypertension and the study sample. Statistically significant values (p < 0.05) are written in bold.**

## Discussion

In the current study, the gender differences in the prevalence and prognostic value of different ECG markers were studied in a large middle-aged general population, and many differences between men and women were found. The prevalence of all endpoints was considerably higher in men than in women. Women had more often a normal electrocardiogram compared to men. Electrocardiographic LVH seemed to have different prognostic value between middle-aged men and women.

The prevalence of all endpoints in the current study were considerably higher among men which is in line with the previous reports (Kannel et al., 1998; Albert et al., 2003; Haukilahti et al., 2019). The risk of CAD increases considerably with age among both genders (Jousilahti et al., 1999). Approximately 40% of the subjects with cardiac death or SCD had CAD, and the relatively low proportion of CAD can be largely explained with the mean age of the study population. The proportion of CAD was expectedly higher among male subjects with cardiac endpoint. Both CAD (Airaksinen et al., 2016) and SCD (Kannel et al., 1998) have been shown to occur approximately 10 years later in women than in men. However, the prevalence of CVD among women has been reported to equal that of men with advancing age (Maffei et al., 2019). In addition, the prevalence of any vascular disease has been shown to be higher with each decade of life, and each additional decade of life approximately doubles the risk of vascular disease (Savji et al., 2013).

The presence of any ECG abnormality was significantly more commonly found among male subjects who had experienced cardiac death in comparison to their female counterparts. Two fifths of the female cardiac death victims did not have any prior ECG abnormalities while approximately a quarter of the male victims had a normal electrocardiogram. The difference in the proportion of normal electrocardiograms narrowed in SCD victims, yet the gender difference still existed.

In the present study, ECG signs of LVH was found to be more prevalent among men who had experienced cardiac death, yet the overall number of subjects with ECG sign of LVH was low in both genders leading to lack of statistical power in the multivariate model. However, in the gender interaction analysis the prognostic value of LVH for cardiac death and all-cause mortality was stronger in women than in men. The risk of cardiovascular events among asymptomatic subjects is 1.6-fold in subjects with LVH, and slightly higher among women than in men (Chou et al., 2011). Previously, LVH has also been associated with the risk of cardiovascular mortality (Prineas et al., 2001). In a study by Desai et al. (2012), the risk of cardiovascular mortality was over eightfold among women without clinical CVD but the presence of ECG sign of LVH defined by criteria of Sokolow-Lyon, and nearly fivefold in men. In addition, the risk of adverse outcomes was found to be higher in each subgroup and among both genders if LVH co-existed with repolarization abnormalities. Previously, in a large middle aged general population cohort the ECG sign of LVH observed with strain pattern was associated with over sixfold risk for CAD event in white men and over twofold risk in black women (Machado et al., 2006). In a current study the general population sample was even larger than in those two studies with similar kind of findings. In the present study LVH with inferolateral T wave inversion associated with increased relative risk for cardiac death in men, yet the number of cases was limited. Previously, T-wave inversions and changes in ST segment with ECH LVH are associated with more severe LVH and elevated risk of adverse outcomes (Hancock et al., 2009; Rautaharju et al., 2009; Bang et al., 2014).

Prolonged QRS duration is a well-known marker of adverse prognosis in subjects with cardiac disease (Kashani and Barold, 2005; Schinkel et al., 2009) but also in the general population (Aro et al., 2011). However, the previous literature on sex differences in prognostic value of QRS prolongation is sparse. In the current study, prolonged QRS duration was more prevalent among male subjects who experienced cardiac death and it was associated with higher relative risk for cardiac death among men than among women. Other known risk factors for cardiovascular events are changes in ST segment and T-wave (Chou et al., 2011). In the current study we did not assess ST segment changes alone. T-wave inversions were slightly more commonly found among women than in men who had experienced cardiac death, yet they remained statistically significant in a multivariate model for this endpoint only among men. Previously T-wave abnormalities have been associated with 1.6–2.1 -fold risk for CAD in the general population (Machado et al., 2006; Chou et al., 2011) and inferolateral T-wave inversions also to increased risk for cardiac death and SCD (Aro et al., 2012; Laukkanen et al., 2014). However, the information of gender differences in prognostic value of T wave inversions is sparse. In a current study, T-wave inversions and LVH remained significant predictors of SCD in multivariable analysis also in women. No gender interaction was seen in any of the variables as predictors of SCD. These data show that ECG may be even better independent predictor of more specific modes of death, such as SCD, in women. Cardiac death can be due to many different mechanisms, which may explain why it is not well predicted by standard ECG, especially in women.

We pooled the data from three different population samples, because the event rates in single samples are relatively low in women. This is an obvious limitation of the study. The study populations represent the Finnish general population from different decades and treatments for cardiac diseases have developed dramatically during the follow-up period. On this account all results might not be completely transferable to current decade even though a quarter of the population was studied in the twenty-first century. It is also possible that the health behavior has changed considerably during the decades. However, the populations were quite homogenous in terms of cardiovascular risk factors. The lack of other ethnicities may also be considered as a limitation of our study. Regarding the ECG variables determined in this study, standard QTc limits for men and women were used (Rautaharju et al., 2009), but there is no uniformly accepted gender-specific limits for QRS duration, even though there is a subtle difference in QRS duration between men and women. Therefore, QRS > 110 ms was used as a cut-off point in the study and the selected cut off point in the study and the selected cut off point was based on one large retrospective register study (Desai et al., 2006). In addition, LVH could have been more definitely diagnosed by echocardiography, which was not available at the start of the collection of these populations. However, recently ECG LVH has supposed to be at least partly distinct entity from echocardiographic LVH (Aro and Chugh, 2016) and also to be independently prognostic for cardiovascular mortality and morbidity with similar level as LVH diagnosed by magnetic resonance imaging (Bacharova et al., 2015). To our knowledge, this is the largest study which has been conducted to determine the prevalence and prognostic significance of multiple ECG variants for cardiac mortality, SCD and all-cause mortality in the middle-aged general population. Still, the event rate in SCD, especially among women, remains low due to the nature of SCD which causes challenges in obtaining enough statistical power when performing gender stratified analysis.

In conclusion, we studied the impact of gender on the prevalence and prognostic value of different ECG markers in a large middle-aged general population. All mortality endpoints were more commonly found among male subjects. The prevalence of all ECG variables apart from fQRS was higher among men in comparison to women. Based on this study, electrocardiographic LVH has a slightly different prognostic value between middle-aged men and women, with more pronounced prognostic value for cardiac death among women. Similarly, T-wave inversions, prolonged QRS duration and inferolateral ER were associated with increased risk of cardiac death only among men.

## Data Availability Statement

Publicly available datasets were analyzed in this study. This data can be found here: https://thl.fi/.

## Ethics Statement

The studies involving human participants were reviewed and approved by the Ethics Committee of Northern Ostrobothnia Hospital District. Written informed consent for participation was not required for this study in accordance with the national legislation and the institutional requirements.

## Author Contributions

HH and MH: conceptualization. AE, AH, MH, PK, HR, MJ, TK, JT, AA, and OA: data curation. MH and TK: investigation, methodology, and visualization. HH and MJ: project administration and supervision. TK: software. MH: writing—original draft. TK, HH, MJ, AE, AH, PK, HR, JT, AA, and OA: writing—review and editing. All authors contributed to the article and approved the submitted version.

## Conflict of Interest

The authors declare that the research was conducted in the absence of any commercial or financial relationships that could be construed as a potential conflict of interest.

## Abbreviations

BMI: body mass index
CAD: coronary artery disease
CI: confidence interval
CVD: cardiovascular disease
ECG: electrocardiographic
ER: early repolarization
fQRS: fragmented QRS complex
HR: hazard ratio
LVH: left ventricular hypertrophy
SCD: sudden cardiac death
SD: standard deviation.
